# The long-term survival outcomes of gastric cancer patients with total intravenous anesthesia or inhalation anesthesia: a single-center retrospective cohort study

**DOI:** 10.1186/s12885-021-08946-7

**Published:** 2021-11-10

**Authors:** Wei-Wei Wu, Wei-Han Zhang, Wei-Yi Zhang, Kai Liu, Xin-Zu Chen, Zong-Guang Zhou, Jin Liu, Tao Zhu, Jian-Kun Hu

**Affiliations:** 1 Department of Anesthesiology, West China Hospital, Sichuan University, No. 37 Guo Xue Street, Chengdu, Sichuan Province China; 2 Department of Gastrointestinal Surgery and Laboratory of Gastric Cancer, State Key Laboratory of Biotherapy, West China Hospital, Sichuan University, and Collaborative Innovation Center for Biotherapy, No. 37 Guo Xue Street, Chengdu, Sichuan Province China

**Keywords:** Gastric cancer, Anesthesia, Intravenous, Inhalation, Prognosis

## Abstract

**Background:**

The relationship between the type of anesthesia and the survival outcomes of gastric cancer patients is uncertain. This study compared the overall outcome of gastric cancer patients after surgery with total intravenous anesthesia (TIVA) or inhalation anesthesia (IHA).

**Methods:**

Clinicopathological variables of gastric cancer patients were retrieved from the database of the Surgical Gastric Cancer Patient Registry in West China Hospital, Sichuan University. Patients were grouped according to whether they received TIVA or IHA during the operation. Propensity score (PS) matching was used to balance the baseline variables, and survival outcomes were compared between these two groups. In addition, studies comparing survival outcomes between TIVA and IHA used for gastric cancer surgery and published before April 20th, 2020, were identified, and their data were pooled.

**Results:**

A total of 2827 patients who underwent surgical treatment from Jan 2009 to Dec 2016 were included. There were 323 patients in the TIVA group and 645 patients in the IHA group, with 1:2 PS matching. There was no significant difference in overall survival outcomes between the TIVA and IHA groups before matching the cohort (*p* = 0.566) or after matching the cohort (*p* = 0.679) by log-rank tests. In the Cox hazard regression model, there was no significant difference between the TIVA and IHA groups before (HR: 1.054, 95% CI: 0.881–1.262, *p* = 0.566) or after (HR: 0.957, 95% CI: 0.779–1.177, *p* = 0.679) PS matching. The meta-analysis of survival outcomes between the TIVA and IHA groups found critical statistical value in the before PS matching cohort (HR 0.74, 95% CI: 0.57–0.96 *p* < 0.01) and after PS matching cohort (HR: 0.65, 95% CI: 0.46–0.94, p < 0.01).

**Conclusions:**

Combined with the results of previous studies, total intravenous anesthesia has been shown to be superior to inhalation anesthesia in terms of overall survival for gastric cancer patients undergoing surgical treatment. The selection of intravenous or inhalation anesthesia for gastric cancer surgery should take into account the long-term prognosis of the patient.

**Supplementary Information:**

The online version contains supplementary material available at 10.1186/s12885-021-08946-7.

## Introduction

Gastric cancer is one of the most common malignant diseases of the digestive system, especially in East Asian countries [1, 2]. Radical surgical treatment with perioperative chemotherapy is the major treatment choice according to the latest treatment guidelines [3, 4]. Several clinicopathological variables, such as macroscopic type, Lauren classification, differentiation degree of the tumor, tumor stage, resection degree, resection patterns, and lymphadenectomy degree, are independent prognostic factors of gastric cancer patients [5, 6].

In the perioperative period, both surgical stress and anesthetics may influence cell-mediated immunity and humoral immunity by influencing the functions of immune competent cells and inflammatory mediator secretion, resulting in immunosuppression. Meanwhile, immunosuppression attributable to anesthetics may accelerate the growth and metastases of cancer cells and result in poor survival of patients with malignant diseases [7]. In addition, anesthetics were found to suppress the activity of natural killer cells and promote tumor metastasis in rat models [8]. Anesthetics can also result in immunological suppression by influencing the function of natural killer cells, as shown in a clinical study of breast cancer patients [9].

Specifically, propofol-based total intravenous anesthesia has been found to have fewer immunosuppressive effects than sevoflurane-based or desflurane-based inhalation anesthesia. Previous studies have shown that survival outcomes are significantly better in prostate cancer and colon cancer patients who receive propofol-based total intravenous anesthesia than in those who receive desflurane-based inhalation anesthesia [10, 11]. However, the debate regarding the influence of anesthesia types on the long-term survival outcomes of patients with malignant disease has not been settled. For example, some studies reported no relevance of the type of anesthesia for the prognosis of patients with breast cancer [12, 13]. However, paradoxical survival outcomes have been reported between total intravenous anesthesia and inhalation anesthesia in gastric cancer patients [14–16].

Therefore, we performed this single-center retrospective cohort study with a large sample size and adjusted for the clinicopathological prognostic characteristics by the propensity score (PS) matching method between total intravenous anesthesia (TIVA) and inhalation anesthesia (IHA). The purpose of this study was to assess the relationship between types of anesthesia and long-term overall survival outcomes after gastric cancer surgery.

## Methods

### Data available

We collected data from the database of the Surgical Gastric Cancer Patient Registry in West China Hospital with the registration number WCH-SGCPR-2020. The establishment of this database was approved by the Biomedical Ethical Committee of the West China Hospital, Sichuan University, China (No. 2014–215). Patient records and personal information were deidentified before statistical analysis.

**Fig. 1:**
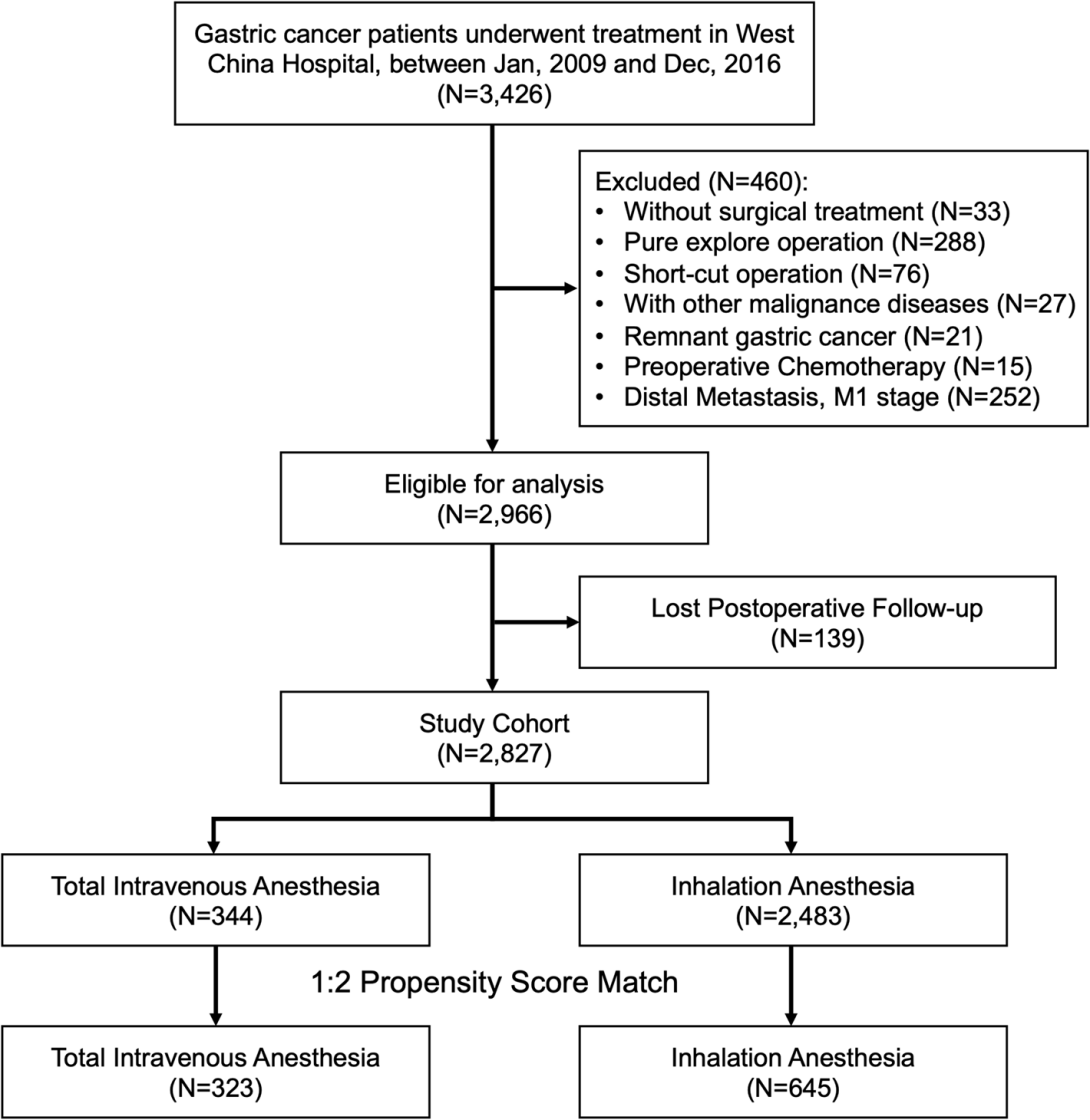
Flow chart of patients’ selection

Patients who underwent surgical treatment from Jan 1st, 2009 to Dec 31st, 2016 in the WCH-SGCPR database were screened [17, 18]. We included primary gastric cancer patients who underwent surgical resection, with complete intraoperative anesthesia information and postoperative follow-up information (updated to Jan 1st, 2020). Patients with a history of other malignant diseases, preoperative chemotherapy or radiotherapy were excluded from the present study. The selection of the patients is presented in Fig. 1.

### Anesthesia method

The choice of anesthesia type was determined by the characteristics of the patients and the preference of the responsible anesthesiologists, and usually choose the type they are better at. In the present study, the patients were divided into the TIVA group and the IHA anesthesia group according to the anesthesia methods. In both the TIVA and IHA groups, anesthesia was induced with midazolam 0.05–0.15 mg/kg, 0.3 μg/kg sufentanil, and 1–2.5 mg/kg propofol. The maintenance dose of anesthesia was propofol 3 mg/(kg*h) or sevoflurane 1 ~ 2% and remifentanil 0.1 ~ 0.2 μg/(kg*min). For patients with total intravenous anesthesia, anesthesia was maintained with propofol and remifentanil infusion. For patients with inhalation anesthesia, anesthesia was maintained with sevoflurane or desflurane inhalation and remifentanil infusion. Patient-controlled analgesia was recommended for all gastric cancer patients who underwent surgical treatment in our hospital and they received a total dose of 3 μg/mL fentanyl or 0.5 μg/mL sufentanil for 72–120 h postoperatively. Nonsteroidal anti-inflammatory drugs (NSAIDs), flurbiprofen axetil or parecoxib sodium were used as rescue solutions during the postoperative recovery period and the hospital stay.

### Surgical treatment method

All of the included patients underwent surgical treatment in the Department of Gastrointestinal Surgery, West China Hospital, Sichuan University. A radical operation with curative intent was performed according to the Japanese Gastric Cancer Treatment Guidelines [3]. Total or subtotal gastrectomy was performed according to the tumor stage, tumor location and status of the regional lymph nodes. Intraoperative frozen sections were routinely performed to secure safe resection margins. There were no limitations on the reconstruction methods.

### Clinicopathological characteristics

The following clinicopathological information was also retrieved from the database: age (years), sex (male or female), tumor size (cm), Borrmann type (Type I-IV), differentiation degree (well, moderate, poor and undifferentiated), tumor location (adenocarcinoma of the esophagogastric junction (AEG) and non-AEG), operation type (laparoscopic surgery and open surgery), radical degree (R0, R1, and R2), lymphadenectomy degree (D1, D1+, D2, and D2+), operation time (minutes), blood loss (ml), pathological tumor stage (pT, pN and pTNM), number of positive and examined lymph nodes, postoperative nonsteroidal anti-inflammatory drug (NSAID) use, adjuvant chemotherapy and postoperative recovery course (complications and hospital stay). For the clinicopathological variables, the pathological examination was performed by pathologists of the Department of West China Hospital, Sichuan University according to the AJCC 8th staging manual [19].

### Follow-up information

Postoperative follow-up was scheduled for each gastric cancer patient who underwent treatment in our department. We recommended at least two outpatient follow-ups in the first 3 years and at least one outpatient follow-up in subsequent years. At each outpatient visit, a physical examination, serum tumor markers (CEA, CA19–9, CA125, CA72–4), and enhanced computed tomography (chest and abdominal) were essential tests. Follow-up information was updated to Jan 1st, 2020. The main reasons for patients lost to follow-up were refused to attend the outpatient visits or changes in contact information. Of the 2966 patients eligible for analysis, 139 patients were lost to postoperative follow-up, so the follow-up rate was 95.3% (2827/2966), with a 48.8 (23.3–77.4) month median follow-up duration.

### Meta-analysis between intravenous and inhalation anesthesia methods of gastric cancer surgery

A comprehensive literature search was performed in the Cochrane Library (January 1, 2005 to November 25, 2020), MEDLINE via PubMed (January 1, 1966 to November 25, 2020) and EMBASE (January 1, 1974 to December 02, 2020) using the terms “gastric cancer”, “gastric carcinoma”, “gastric neoplasm”, “stomach cancer”, “stomach carcinoma”, “stomach neoplasm”, “inhalation, anesthesia”, “insufflation”, “volatile”, “intravenous, anesthesia”, “infusion”, “surgery” and “operation”. Previously published meta-analyses and systematic reviews were also searched for relevant articles. Relevant articles were also retrieved by manually checking the reference lists of the retrieved articles. Titles, abstracts, and subsequently full-text articles were screened by two authors (WW Wu and WH Zhang). We only included studies comparing survival outcomes between total intravenous anesthesia and inhalation anesthesia methods of gastric cancer surgery. Review articles, case reports, articles in languages other than English, and articles with incomplete or duplicated data were excluded.

Data from the included studies were independently extracted by two authors (WH Zhang, WW Wu). For each study, we recorded the name of the first author, year of publication, country, study design, and period of the included patients. The following variables were also extracted: age (mean ± SD), sex, tumor stage and survival outcomes (hazard ratio, HR; 95% confidence intervals, 95% CI). The meta-analysis of survival outcomes was performed by the random-effects method according to the Cochrane guidelines. We evaluated all included studies for quality with the Newcastle-Ottawa Scale (NOS), and all studies were rated a minimum of 5 points.

### Statistical analysis

Continuous variables with a non-normal distribution are expressed as the median and interquartile range (IQR, 25–75%); categorical variables are expressed as numbers (%). The Mann–Whitney U test was used to analyze continuous variables and ordinal categorical variables, whereas the chi-square test was used for unordered categorical variables. Variables that yielded *p* < 0.1 in univariate survival analysis were considered candidates in the multivariate Cox-hazard model. A *P* value< 0.05 (2-sided) was defined as statistically significant.

For patients in the TIVA group, propensity scores were computed as the conditional probability using a logistic regression model that included baseline characteristics (age, sex, tumor location, operation type, radical degree, lymphadenectomy degree, pT stage, pN stage, pM stage, and adjuvant chemotherapy) to achieve balance in covariates between the TIVA and IHA groups. Propensity score matching pairs were identified without replacement using a 1:2 nearest neighbor matching algorithm with caliper width determined by the recommendation (0.001 of the standard deviation of the logit) [20]. The balance of covariates between the TIVA and IHA groups was assessed by the standardized mean difference (SMD). An SMD < 0.1 indicated a good balance in the covariates between the two groups. All statistical analyses were conducted using R Software (http://www.R-project.org/), including the “survival”, “survminer”, “ggplot2”, “nonrandom”, “MatchIt”, “meta” and “metafor” packages.

Considering that this is a retrospective study, we calculated the statistical power via PASS 11 (Version 11.0.7).

## Results

### Patient characteristics before and after propensity score matching

**Fig. 2:**
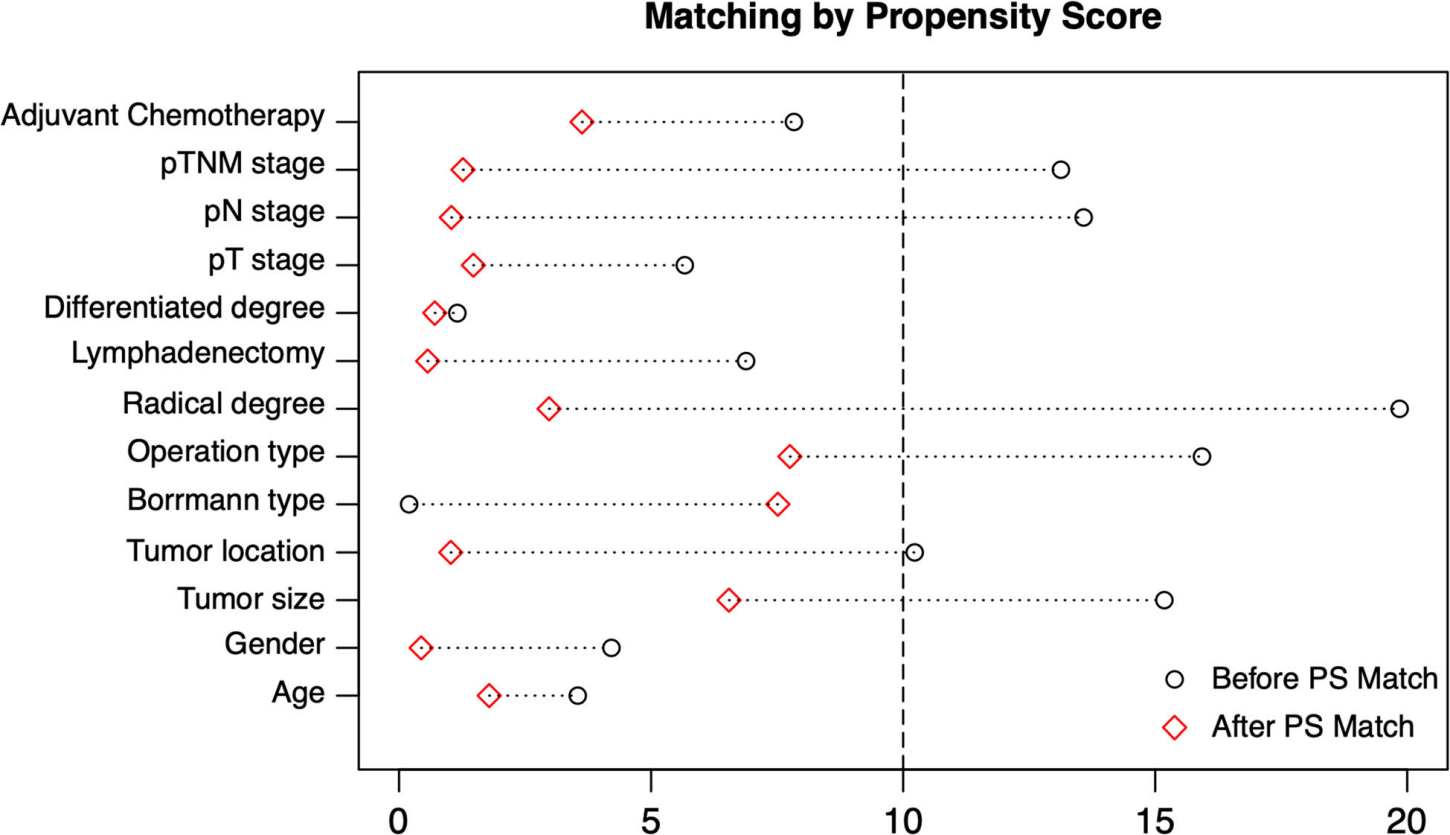
Illustration of standardized differences in clinicopathological characteristics before and after propensity-score matching cohorts

A total of 3426 patients underwent treatment in the Department of Gastrointestinal Surgery, West China Hospital, Sichuan University from January 2009 to December 2016. According to the inclusion criteria and exclusion criteria, 2827 patients were included in the final analysis, 344 patients with total intravenous anesthesia were in the TIVA group, and 2483 patients with inhalation anesthesia were in the IHA group. The clinicopathological and intraoperative characteristics of the patients were compared between the TIVA and IHA groups before and after propensity score matching, and the results are presented in Table 1. Before PS matching, tumor size, operation type, radical degree, blood loss, pTNM stage, numbers of examined, positive lymph nodes were unbalanced between the TIVA and IHA groups (*p* < 0.05 and SMD > 0.1). After the 1:2 PS matching procedures, 323 patients in the TIVA group and 645 patients in the IHA group had balanced clinicopathological covariates (*p* > 0.05 and SMD ≤ 0.1). The standardized differences and distribution of the clinicopathological characteristics before and after propensity score matching are shown in Fig. 2.

**Table 1:** Clinicopathological characteristics between TIVA group and IHA group, before and after propensity-score match

Characteristics		TIVA group *N* = 323 (%)	IHA group *N* = 2264 (%)	*P* value	SMD	TIVA group *N* = 344 (%)	IHA group *N* = 688 (%)	*P* value	SMD
Age, median (IQR)	Years	60.0 [51.0, 66.0]	59.00 [50.0, 66.0]	0.628	0.035	60.0 [51.0, 66.0]	60.0 [51.0, 67.0]	0.967	0.018
Gender	Female	102 (31.6)	671 (29.6)	0.517	0.042	102 (31.6)	205 (31.8)	1	0.004
Tumor Size, median (IQR)	cm	4.0 [3.0, 6.0]	5.0 [3.0, 6.0]	0.012	0.152	4.0 [3.0, 6.0]	4.0 [3.0, 6.0]	0.435	0.065
Borrmann Type	Type III-IV	131 (40.6)	916 (40.5)	1	0.002	131 (40.6)	238 (36.9)	0.301	0.075
Differentiated Degree	G3-G4	260 (80.5)	1812 (80.0)	0.905	0.012	260 (80.5)	521 (80.8)	0.986	0.007
Tumor Location	Non-AEG	74 (22.9)	619 (27.3)	0.106	0.102	74 (22.9)	145 (22.5)	0.945	0.010
Operation Type	Laparoscopic	29 (9.0)	318 (14.0)	0.016	0.159	29 (9.0)	73 (11.3)	0.314	0.078
Radical Degree	R1/R2	4 (1.2)	103 (4.5)	0.008	0.198	4 (1.2)	6 (0.9)	0.912	0.03
Lymphadenectomy Degree	D2/D2+	291 (90.1)	1991 (87.9)	0.303	0.069	291 (90.1)	580 (89.9)	1	0.006
Operation Time, median (IQR)	min	230.0 [205.0, 260.0]	235.0 [205.0, 270.0]	0.368	0.067	230.0 [205.0, 260.0]	230.0 [200.0, 265.0]	0.928	0.007
Blood Loss, median (IQR)	ml	100.0 [50.0, 105.0]	100.0 [80.0, 150.0]	0.032	0.147	100.0 [50.0, 105.0]	100.0 [80.0, 200.0]	0.088	0.126
pT stage	T1	68 (21.1)	491 (21.7)	0.242	0.120	68 (21.1)	140 (21.7)	0.515	0.102
	T2	57 (17.6)	328 (14.5)			57 (17.6)	126 (19.5)		
	T3	73 (22.6)	460 (20.3)			73 (22.6)	120 (18.6)		
	T4	125 (38.7)	985 (43.5)			125 (38.7)	259 (40.2)		
pN stage	N0	126 (39.0)	771 (34.1)	0.125	0.143	126 (39.0)	256 (39.7)	0.917	0.048
	N1	60 (18.6)	372 (16.4)			60 (18.6)	108 (16.7)		
	N2	52 (16.1)	417 (18.4)			52 (16.1)	106 (16.4)		
	N3	85 (26.3)	704 (31.1)			85 (26.3)	175 (27.1)		
pTNM stage	I	92 (28.5)	575 (25.4)	0.026	0.161	92 (28.5)	189 (29.3)	0.962	0.019
	II	91 (28.2)	529 (23.4)			91 (28.2)	178 (27.6)		
	III	140 (43.3)	1160 (51.2)			140 (43.3)	278 (43.1)		
No. of Positive LNs, median (IQR)	numbers	2.0 [0.0, 7.0]	2.0 [0.0, 8.0]	0.013	0.138	2.0 [0.0, 7.0]	2.0 [0.0, 7.0]	0.806	0.034
No. of Examined LNs, median (IQR)	numbers	26.0 [19.5, 35.0]	27.0 [20.0, 37.0]	0.065	0.159	26.0 [19.5, 35.0]	26.0 [20.0, 36.0]	0.670	0.066
Perioperative NSAIDs	Use	6 (1.9)	57 (2.5)	0.598	0.045	6 (1.9)	16 (2.5)	0.701	0.043
Adjuvant Chemotherapy	Yes	183 (56.7)	1370 (60.5)	0.207	0.078	183 (56.7)	377 (58.4)	0.643	0.036
Postoperative 30-day complications	Yes	54 (16.7)	411 (18.2)	0.582	0.038	54 (16.7)	114 (17.7)	0.779	0.025
Postoperative Hospital Stay, median (IQR)	Days	10.00 [9.00, 12.00]	10.00 [9.00, 12.00]	0.992	0.031	10.00 [9.00, 12.00]	10.00 [9.00, 12.00]	0.833	0.015

Abbreviations: *TIVA* Total intravenous anesthesia, *IHA* Inhalation anesthesia, *SMD* Standardized mean difference, *IQR* Interquartile range, *AEG* Adenocarcinoma of esophagogastric junction, *LNs* Lymph nodes

### Univariate and multivariate survival analysis

**Fig. 3:**
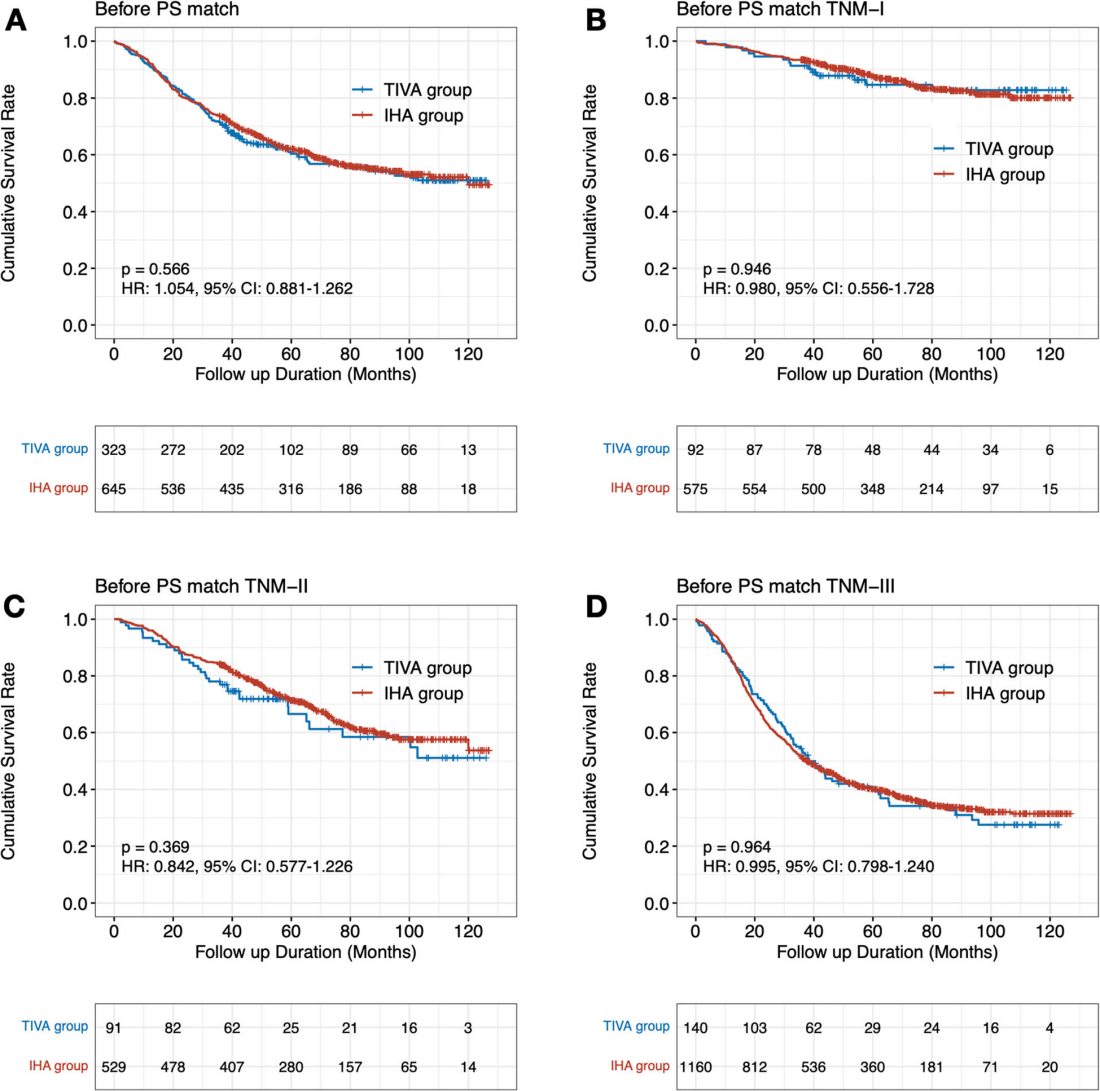
Survival outcomes between TIVA and IHA groups and subgroup analyses before propensity-score matching cohorts (A. Survival rate for all TNM stages; B. Survival rate for TNM-I stage; C. Survival rate for TNM-II stage; D. Survival rate for TNM-III stage)

**Fig. 4:**
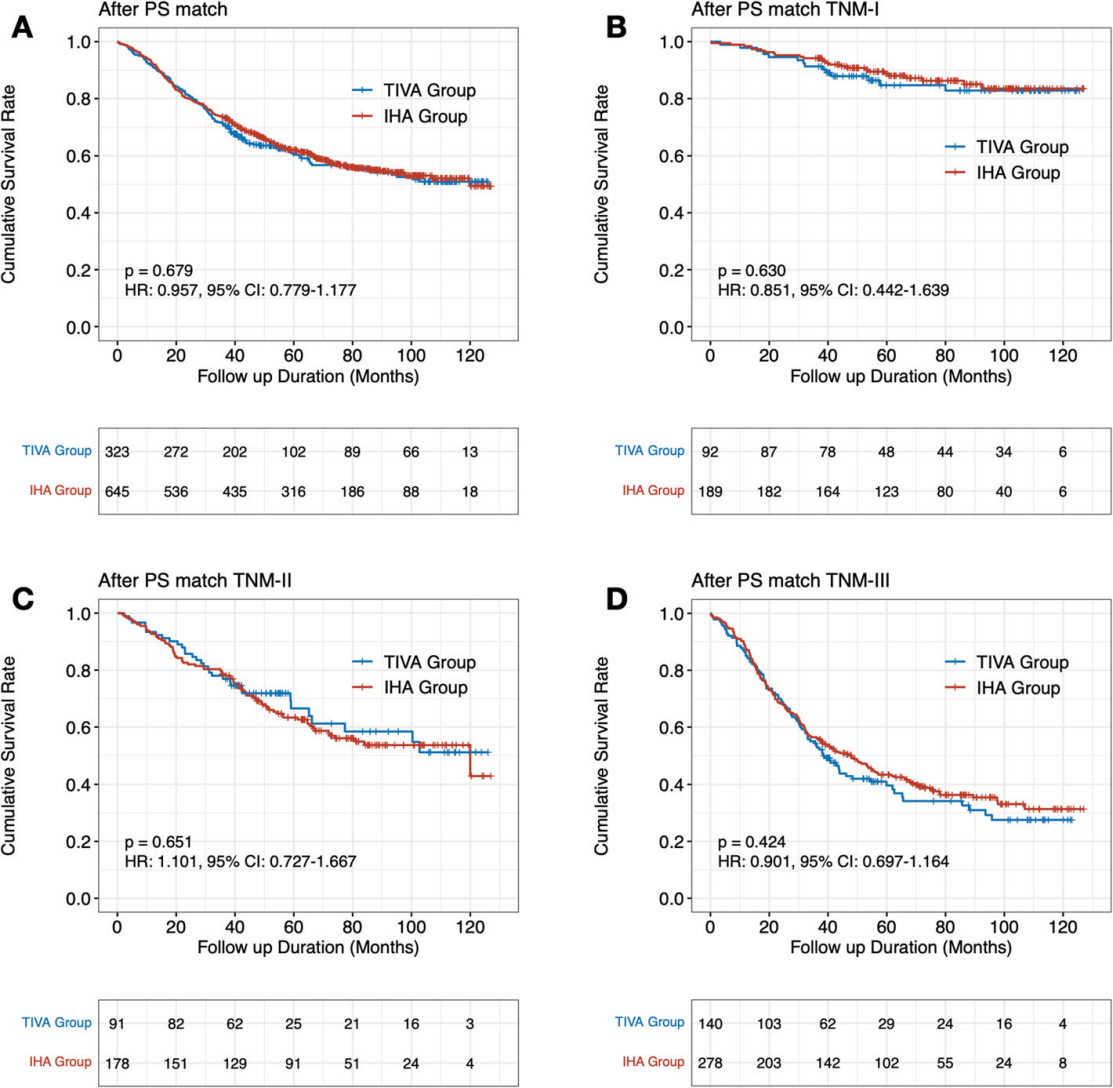
Survival outcomes between TIVA and IHA groups and subgroup analyses in after propensity-score matching cohorts (A. Survival rate for all TNM stages; B. Survival rate for TNM-I stage; C. Survival rate for TNM-II stage; D. Survival rate for TNM-III stage)

First, we evaluated the survival outcomes between the TIVA and IHA groups, and there was no survival difference by the log-rank test between the TIVA and IHA groups before PS matching (HR: 1.054, 95% CI: 0.881–1.262, *p* = 0.566) and after PS matching (HR: 0.957, 95% CI: 0.779–1.177, *p* = 0.679) (Figs. 3 and 4).

Univariate and multivariate survival analyses of patients in the before and after PS matching cohorts were analyzed and are presented in Tables 2 and 3, respectively. In the multivariate survival analysis of before propensity score matching cohorts, age, tumor size, macroscopic type, radical degree, pathological TNM stage, and postoperative adjuvant chemotherapy were independent prognostic risk factors for overall survival. Additionally, in the multivariate survival analysis of patients after the propensity score matching cohort, age, tumor size, radical degree, pathological TNM stage, and adjuvant chemotherapy were independent prognostic risk factors for the overall survival outcomes. Most importantly, the anesthesia type (intravenous anesthesia or inhalation anesthesia methods) was not a significant risk factor for overall survival outcomes in either the before (HR: 1.054, 95% CI: 0.881–1.262, *p* = 0.566) or after propensity score matching cohorts (HR: 0.957, 95% CI: 0.779–1.177, *p* = 0.679).

**Table 2:** Univariate and Multivariate survival analysis of patients before propensity-score match (*N* = 2587)

Characteristics		Univariate			Multivariate		
		HR	95% CI	*P* value	HR	95% CI	*P* value
Age, years	< 65 vs. ≥65	1.307	1.157–1.477	< 0.001	1.217	1.075–1.379	0.002
Gender	Male vs. Female	0.998	0.879–1.133	0.978			
Tumor location	AEG vs. Non-AEG	0.723	0.638–0.819	< 0.001	0.882	0.777–1.002	0.054
Tumor size, cm	< 5 vs. ≥5	2.714	2.388–3.084	< 0.001	1.352	1.168–1.566	< 0.001
Macroscopic type	Type 0–2 vs. Type 3–4	2.080	1.850–2.338	< 0.001	1.164	1.026–1.321	0.019
Differentiate degree	G1–2 vs. G3	1.517	1.291–1.782	< 0.001	1.105	0.935–1.306	0.240
Radical degree	R0 vs. R1/R2	3.084	2.473–3.846	< 0.001	1.920	1.534–2.404	< 0.001
Lymphadenectomy degree	D1/D1+ vs. D2/D2+	1.035	0.869–1.232	0.701			
pTNM stage	I vs. II	2.601	2.048–3.304	< 0.001	2.316	1.803–2.974	< 0.001
	I vs. III	6.519	5.284–8.042	< 0.001	4.970	3.916–6.308	< 0.001
Adjuvant chemotherapy	No vs. Yes	0.884	0.786–0.995	0.041	0.715	0.634–0.807	< 0.001
Anesthesia method	IVA vs. IHA	1.054	0.881–1.262	0.566	0.932	0.778–1.116	0.441
NSAIDs	No vs. Yes	1.183	0.828–1.691	0.355			

Abbreviations: *HR* Hazard ratio, *CI* Confidence interval, *AEG* Adenocarcinoma of esophagogastric junction, *TIVA* Total intravenous anesthesia, *IHA* Inhalation anesthesia, *NSAIDs* Nonsteroidal Anti-inflammatory Drugs

**Table 3:** Univariate and Multivariate survival analysis of patients after propensity-score match (*N* = 1032)

Characteristics		Univariate			Multivariate		
		HR	95% CI	*P* value	HR	95% CI	*P* value
Age, years	< 65 vs. ≥65	1.462	1.198–1.785	< 0.001	1.421	1.159–1.743	0.001
Gender	Male vs. Female	0.955	0.774–1.179	0.669			
Tumor location	AEG vs. Non-AEG	0.783	0.629–0.975	0.029	1.006	0.802–1.261	0.961
Tumor size, cm	< 5 vs. ≥5	2.513	2.052–3.077	< 0.001	1.382	1.100–1.736	0.005
Macroscopic Type	Type 0–2 vs. Type 3–4	1.963	1.615–2.386	< 0.001	1.118	0.903–1.383	0.306
Differentiate Degree	G1–2 vs. G3	1.407	1.077–1.837	0.012	1.12	0.849–1.478	0.422
Radical Degree	R0 vs. R1/R2	2.079	0.985–4.391	0.055	1.367	0.644–2.905	0.416
Lymphadenectomy degree	D1/D1+ vs. D2/D2+	1.220	0.883–1.686	0.229			
TNM stage	I vs. II	3.440	2.383–4.965	< 0.001	3.214	2.197–4.703	< 0.001
	I vs. III	6.691	4.775–9.376	< 0.001	5.569	3.834–8.089	< 0.001
Adjuvant Chemotherapy	No vs. Yes	0.833	0.685–1.013	0.068	0.693	0.567–0.847	< 0.001
Anesthesia method	IVA vs. IHA	0.957	0.779–1.177	0.679	0.946	0.769–1.163	0.597
NSAIDs	No vs. Yes	0.574	0.256–1.285	0.177			

Abbreviations: *HR* Hazard ratio, *CI* Confidence interval, *AEG* Adenocarcinoma of esophagogastric junction, *TIVA* Total intravenous anesthesia, *IHA* Inhalation anesthesia, *NSAIDs* Nonsteroidal Anti-inflammatory Drugs

### Subgroup analysis

We performed subgroup analyses on patients based on their final pathological stage. Patients in both the TIVA and IHA groups had similar survival rates across pTNM stages (Figs. 3 and 4). Before PS match, for whole pTNM stage, the HR was 1.054 (95%CI: 0.881–1.262), the propensity score-adjusted HR was 0.957 (95%CI: 0.779–1.177). For pTNM-I, the HR was 0.980 (95%CI: 0.556–1.728), the propensity score-adjusted HR was 0.630 (95%CI: 0.442–1.639). For pTNM-II, the HR was 0.369 (95%CI: 0.577–1.226), the propensity score-adjusted HR was 1.101 (95%CI: 0.727–1.667). For pTNM-III, the HR was 0.995 (95%CI: 0.798–1.240), the propensity score-adjusted HR was 0.901 (95%CI: 0.697–1.164).

### Meta-analysis between total intravenous and inhalation anesthesia

**Fig. 5:**
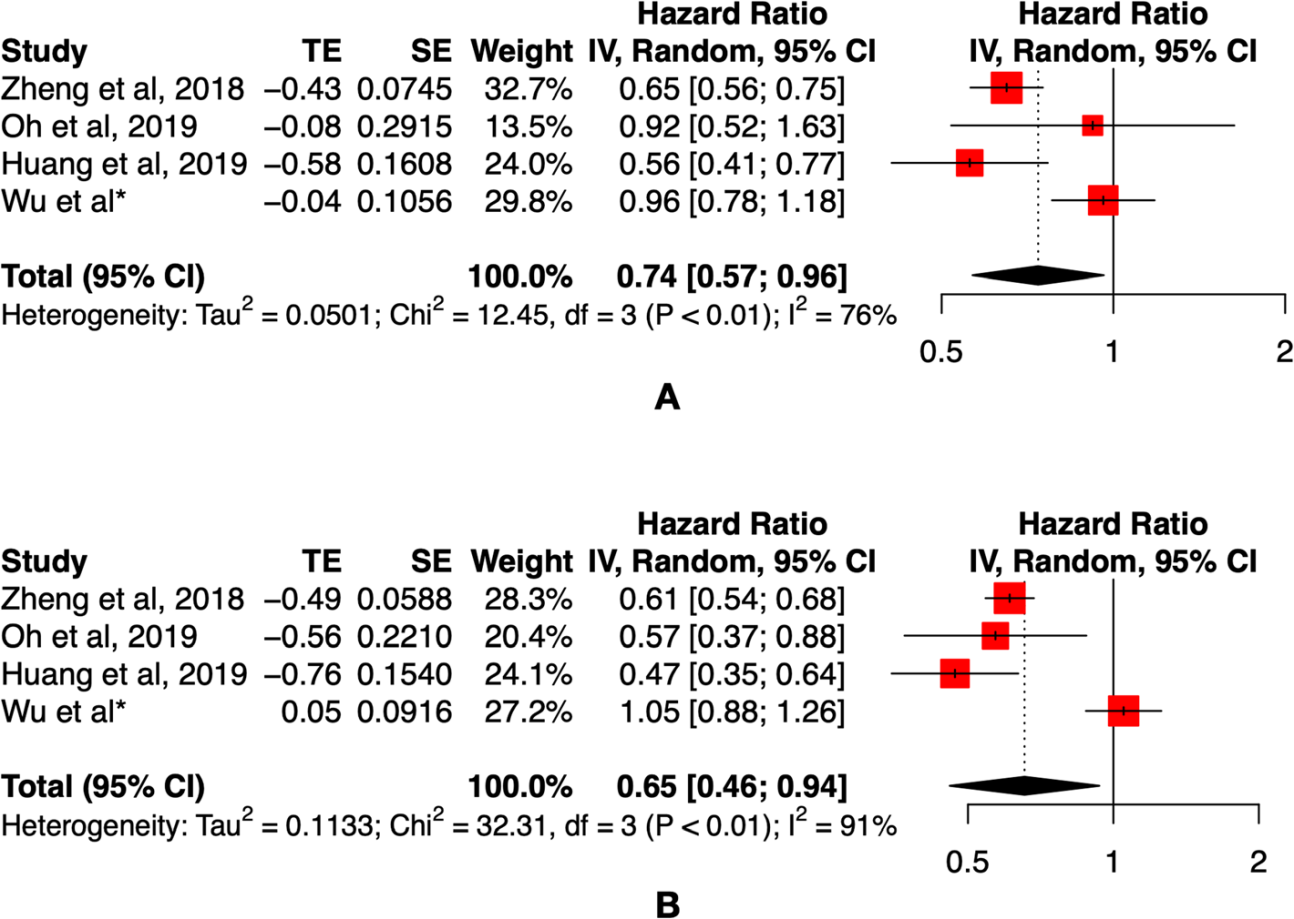
Forest plot of survival outcomes among study compares intravenous anesthesia and inhalation anesthesia (A. Before propensity score matching, B. After propensity score matching)

Through the literature search, we found three published studies that compared the survival outcomes between the TIVA and IHA groups (Supplementary Fig. 1). Meanwhile, we added the survival outcomes of our study to the meta-analysis. The general characteristics of the studies are presented in Table 4. Both of the studies used the propensity score matching method to balance the clinicopathological characteristics between the TIVA and IHA groups. Therefore, a meta-analysis of survival outcomes was performed before and after PS matching (Fig. 5A and B). A critical statistical value was found in the before (HR 0.74, 95% CI: 0.57–0.96, *p* < 0.01) and after (HR 0.65, 95% CI: 0.46–0.94, p < 0.01) PS matching cohort between the TIVA and IHA groups.

**Table 4:** General characteristics of study compare survival outcomes between total intravenous and inhalation anesthesia

Author	Country	Time Period	Tumor Stage	Operation Type	Match method	PS match	No. of Patients		Age (year)		Gender (male)		Survival outcome HR (95% CI)
							TIVA	IHA	IVA	IHA	IVA	IHA	IVA vs. IHA
Oh et al., 2019	Korea	2005–2015	I-III AJCC 7th	Lap and Open	1:1 PS match	Before	816	3791	58.3 ± 12.4	60.5 ± 12.7	564	2511	0.57 (0.37–0.88)
						After	769	769	58.7 ± 12.4	59.3 ± 12.7	527	527	0.92 (0.52–1.63)
Zheng et al., 2018	China	2007–2012	I-III AJCC 7th	Open Surgery	1:1 PS match	Before	1506	1350	NA	NA	313	317	0.61 (0.54–0.68)
						After	897	897	NA	NA	159	160	0.65 (0.56–0.75)
Huang et al., 2019	China	2006–2016	I-IV	Not mentioned	1:1 PS match	Before	190	218	65 ± 14	66 ± 15	124	150	0.47 (0.34–0.63)
						After	167	167	66 ± 14	65 ± 15	114	116	0.56 (0.41–0.78)
Wu et al.*	China	2009–2016	I-III AJCC 8th	Lap and Open	1:2 PS match	Before	323	2264	60.0 [51.0, 66.0]	59.00 [50.0, 66.0]	221	1593	1.05 (0.88–1.26)
						After	323	645	60.0 [51.0, 66.0]	60.0 [51.0, 67.0]	221	440	0.96 (0.78–1.18)

Abbreviations: *TIVA* Total intravenous anesthesia, *IHA* Inhalation anesthesia, *PS* Propensity score, *HR* Hazard ratio, *CI* Confidence interval, *NA* Not applicable

^a^The present study

## Discussion

According to previous studies, conflicting conclusions have been reported on the survival outcomes of total intravenous anesthesia and inhalation anesthesia used during gastric cancer surgery [14–16]. However, in both the before PS matching and after PS matching cohorts, there was no significant difference between TIVA and IHA in the overall survival outcomes of patients who underwent gastric cancer surgery. In the further subgroup analysis, no statistical differences were found in the survival rates of patients at each stage. Meanwhile, we performed a pooled analysis of survival results from previously reported studies and our study to explore whether anesthesia type can influence the survival outcomes of gastric cancer patients who underwent surgical treatment. A critical statistical value was found in the before and after PS matching cohort between the TIVA and IHA groups for overall survival outcomes.

Although other perioperative treatment modalities, such as radiotherapy, may also affect tumor metastasis, the relationship between anesthetic techniques and the prognosis of cancer patients is one of the core concerns during the process of making treatment strategy decisions for malignant diseases. Regional anesthesia, such as epidural anesthesia, has positive implications in the prevention of immunosuppression and in reducing inflammation during the surgical treatment of malignant diseases.

Volatile agents are the most common method of maintaining general anesthesia worldwide. TIVA is primarily administered by propofol as an induction and maintenance agent. Propofol has been shown in in vitro experiments to inhibit the expression of oncogenes and suppress tumor angiogenesis, resulting in a lower recurrence rate [21–24]**.**

The survival impact of anesthetic and anesthesia type used during the operation has been evaluated in several malignant diseases. Total intravenous anesthesia has been found to be associated with significantly better survival outcomes than inhalation anesthesia for esophageal cancer patients [25]. In addition, in a large sample size study of colon cancer patients, total intravenous anesthesia had better survival outcomes than inhalation anesthesia irrespective of the tumor-node-metastasis stage [11]. However, for patients who underwent breast cancer surgery, contradictory results were found in previous studies. A study showed that propofol-based TIVA had a lower tumor recurrence risk than sevoflurane-based IHA for breast cancer patients [26]. However, another existing studies found no significant difference in disease-free survival and overall survival between TIVA and IHA for breast cancer patients [12, 27]. Tumor heterogeneity and molecular characteristics are important explanations for the differential treatment outcomes of cancer patients [28, 29]. Whether these factors can help explain the different results among the different cancer types with total intravenous anesthesia or inhalation anesthesia is unclear. Therefore, not only is there a need for research on the relationship and mechanism of anesthetic drugs and the immunological response, further study is expecting to analyze the effects of anesthetic drugs on the expression levels of oncogenes or the regulation of the tumor microenvironment, corresponding functional changes of tumor biological behavior, and the survival outcomes of cancer patients.

Specific to gastric cancer, only three studies have reported the influence of TIVA or IHA on the survival outcomes of surgical treatment [14–16]. Although these studies both used the PS matching method to balance clinicopathological characteristics, their conclusions were very different. Different tumor stages or surgical treatment strategies might be the reasons for the different survival outcomes between these two studies. For example, in the study of Oh et al., more than half of the patients underwent laparoscopic surgery, whereas patients who underwent laparoscopic surgery were excluded from the study of Zheng et al. [14, 15]. Laparoscopic gastric cancer surgery has a lower risk of adverse inflammatory reactions than open surgery [30]. Therefore, a reduction of adverse immune reactions by a high proportion of laparoscopic surgeries may amplify the effect of inhalation anesthesia on immune suppression.

In our study, we did not exclude laparoscopic surgery and found no survival difference between total intravenous anesthesia and inhalation anesthesia with long-term follow-up. In addition, the different follow-up durations may be another reason for the different survival results of the previous two studies. The limited follow-up duration of the study of Oh et al. may not fully and accurately reflect the survival difference between the TIVA and IHA groups. Our study balanced the operation type (laparoscopic and open operations) and analyzed long-term survival outcomes. Therefore, according to the results of our study, both TIVA and IHA are acceptable anesthesia methods for gastric cancer surgery.

In addition, the contradictory survival results reported for TIVA and IHA used to treat gastric cancers should be considered. We performed a meta-analysis of survival outcomes between total intravenous anesthesia and inhalation anesthesia, including data from previously reported studies and our study. However, there was a significant difference in survival between the TIVA and IHA groups in the meta-analysis. What calls for special attention is that all three studies included in the meta-analysis were retrospective studies, and selection bias and other natural limitations of the retrospective study design cannot be neglected. Therefore, these results have limited reference value for clinical indications of the choice of anesthesia type. According to the present clinical evidence, the selection of total intravenous anesthesia or inhalation anesthesia should be made according to the individualized situation of each patient.

Limitations of all retrospective studies should also be considered when interpreting the results of the present study. First, we adopted the PS matching method, but selection bias in the choice of anesthesia type cannot be neglected. Second, the sample size of the present study was based on the data available during the study period rather than calculated in advance as in a prospective study. There exists the potential of an inadequate sample size and statistical power, which cannot detect a significant difference between the two anesthesia types. Third, recurrence type data were not completely collected in the present study. Therefore, the relationship of TIVA and IHA with recurrence type or recurrence-free survival outcomes was not analyzed. Finally, due to the limitations of the retrospective design, inflammatory markers were not measured, and we could not explain the relationship between the inflammatory response and types of anesthesia used for gastric cancer surgery.

## Conclusions

In conclusion, combined with the results of previous studies, total intravenous anesthesia has been shown to be superior to inhalation anesthesia in terms of overall survival for gastric cancer patients undergoing surgical treatment. The selection of intravenous or inhalation anesthesia for gastric cancer surgery should take into account the long-term prognosis of the patient.

## Supplementary Information

**Additional file 1:** **Supplementary Figure 1.** Flow diagram of study selection (PRISMA format).

## Data Availability

Data supporting the findings of this study are available from the corresponding author upon reasonable request.

## Abbreviations

TIVA: Total intravenous anesthesia
IHA: Inhalation anesthesia
PS: Propensity score
AEG: Adenocarcinoma of the esophagogastric junction
NSAIDs: Nonsteroidal anti-inflammatory drugs
SD: Standard deviation
HR: Hazard ratio
95% CI: 95% confidence intervals
IQR: Interquartile range
PS: Propensity score
SMD: Standardized mean difference

## Declarations

All methods of this study were carried out in accordance with relevant guidelines and regulations.
