# Supplementary figures and images for: The long-term survival outcomes of gastric cancer patients with total intravenous anesthesia or inhalation anesthesia: a single-center retrospective cohort study
Source: BMC Cancer. 2021 Nov 10;21:1193. doi: 10.1186/s12885-021-08946-7 (PMC8579630; doi:10.1186/s12885-021-08946-7)

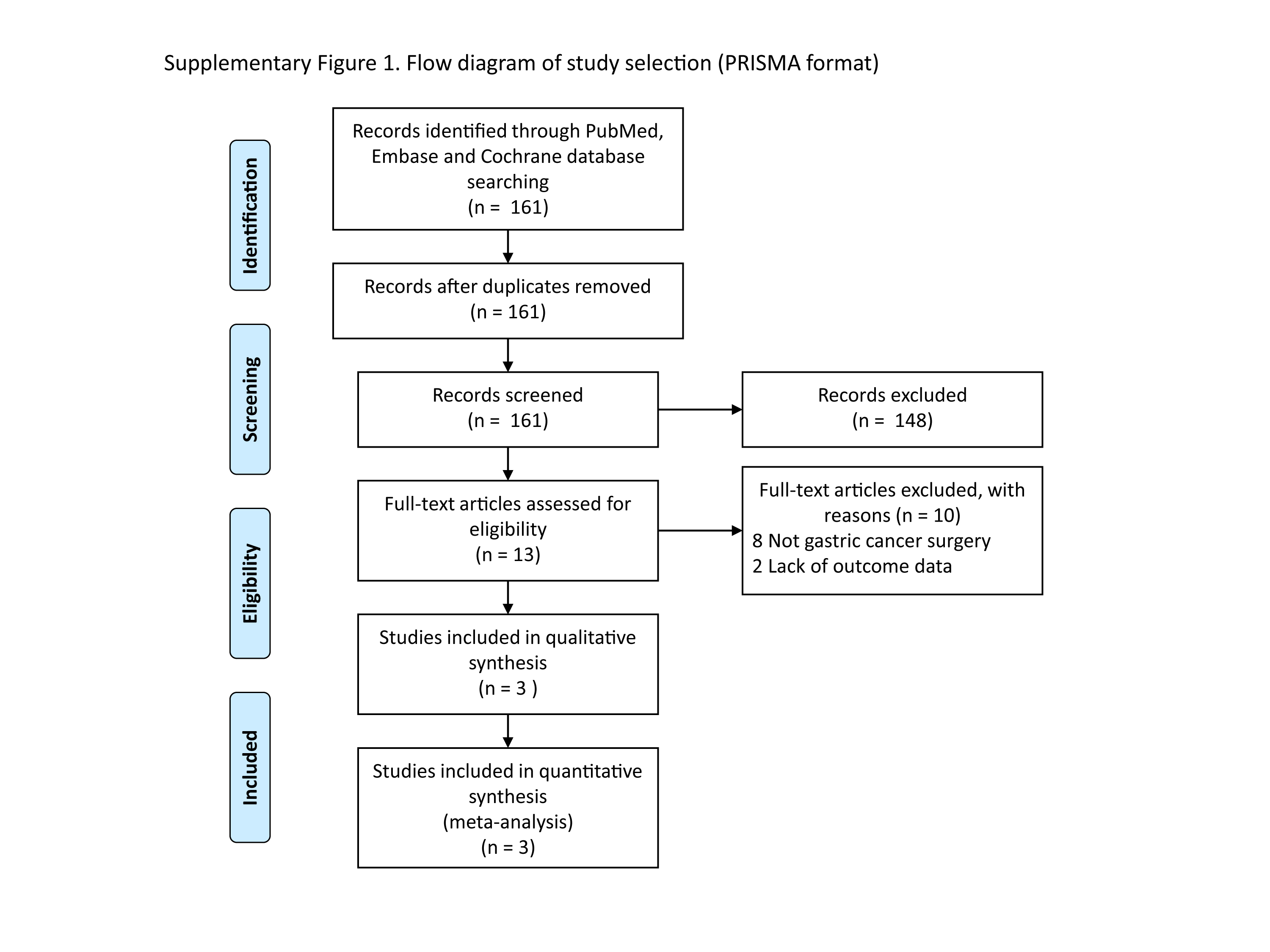

Supplement: Supplementary file 1 — Additional file 1: Supplementary Figure 1. Flow diagram of study selection (PRISMA format). [file 12885_2021_8946_MOESM1_ESM.tif]
